# Factors Associated with Meat Consumption in Students of Spanish Universities: UniHcos Project

**DOI:** 10.3390/ijerph16203924

**Published:** 2019-10-15

**Authors:** Rocío Ortiz-Moncada, María Morales-Suárez-Varela, Ángeles Avecilla-Benítez, Aurora Norte Navarro, Rocío Olmedo-Requena, Carmen Amezcua-Prieto, José M. Cancela, Gemma Blázquez Abellán, Ramona Mateos-Campos, Luis Félix Valero Juan, Susana Redondo Martín, Jéssica Alonso-Molero, Antonio José Molina de la Torre, Agustín Llopis-Morales, Isabel Peraita-Costa, Tania Fernández-Villa, Grupo de Investigación UniHcos

**Affiliations:** 1 Area of Preventive Medicine and Public Health, Department of Community Nursing, Preventive Medicine and Public Health and History of Science, Food and Nutrition Research Group, Universidad de Alicante, Carretera de San Vicente del Raspeig s/n, 03690 San Vicente del Raspeig, Alicante, Spain; rocio.ortiz@ua.es (R.O.-M.); mangabe@gmail.com (Á.A.-B.); 2 Consortium for Biomedical Research in Epidemiology & Public Health (CIBER Epidemiología y Salud Pública-CIBERESP), 28029 Madrid, Spain; rocioolmedo@ugr.es (R.O.-R.); carmezcua@ugr.es (C.A.-P.); ivperaitacosta@hotmail.es (I.P.-C.); 3 Area of Preventive Medicine and Public Health, Department of Preventive Medicine and Public Health, Food Sciences, Toxicology and Legal Medicine, School of Pharmacy, Universitat de Valencia, 46100 Valencia, Spain; agustinllopis@gmail.com; 4 Nursing Department, School of Health Sciences, Universidad de Alicante, Carretera de San Vicente del Raspeig, s/n, 03690 San Vicente del Raspeig, Alicante, Spain; aurora.norte@ua.es; 5 Department of Preventive Medicine and Public Health, Universidad de Granada, 18016 Granada, Spain; 6 Instituto de Investigación Biosanitaria ibs, 15 18071 Granada, Spain; 7 School of Education and Sports Sciences, HealthyFit Research Group, Universidad de Vigo, 36005 Pontevedra, Spain; chemacc@uvigo.es; 8 Area of Preventive Medicine and Public Health, Department of Medical Sciences, School of Pharmacy, Universidad de Castilla-La Mancha, 02071 Albacete, Spain; gemma.Blazquez@uclm.es; 9 Area of Preventive Medicine and Public Health, Department of Biomedical and Diagnostic Sciences, Universidad de Salamanca, 37007 Salamanca, Spain; rmateos@usal.es (R.M.-C.); luva@usal.es (L.F.V.J.); 10 Department of Pathological Anatomy, Microbiology and Preventive Medicine and Public Health, School of Medicine, Universidad de Valladolid, 47005 Valladolid, Spain; redmarsu@jcyl.es; 11 School of Medicine, Universidad de Cantabria, 39005 Santander, Spain; alonsomoleroj@gmail.com; 12 Marqués de Valdecilla Research Institute-IDIVAL, 39011 Santander, Spain; 13 Department of Biomedical Sciences, Area of Preventive Medicine and Public Health, Universidad de León, 24071 León, Spain; ajmolt@unileon.es (A.J.M.d.l.T.); tania.f.v@gmail.com (T.F.-V.); 14 Group of Investigation in Interactions Gene-Environment and Health (GIIGAS)/Institute of Biomedicine (IBIOMED), Universidad de León, 24071 León, Spain

**Keywords:** students, surveys and questionnaires, meat, Mediterranean diet, cross-sectional study

## Abstract

The level of meat consumption is one of the main deviations from the Mediterranean diet pattern in Spanish university students. The objective of this cross-sectional descriptive study is to analyze the association between sociodemographic factors and the consumption of fresh and processed meat in Spanish university students. This study is part of a cohort of 11 Spanish universities with 9862 university students (UniHcos Project). A descriptive analysis and a chi2 test were carried out to assess differences between personal and sociodemographic variables and meat consumption, and binary logistic regression analysis to assess factors associated with consumption; 19.9% and 73.5% met the recommendations for meat-fresh and meat-processed consumption, respectively. Only 3.8% of students meet the recommendations for both fresh and processed meat. Statistically significant differences were found between sex, BMI, employment, housing, and coexistence regarding compliance with recommendations. Female employed students living in rental accommodations with a partner are more likely to meet the recommendations for fresh meats while male, normal weight, employed students living in rental accommodations with a partner are more likely to meet the recommendations for processed meats. There is a lack of compliance with the recommendations for consumption of fresh meat in Spanish university students, differences in compliance among students of differing regions and an association with sex, employment, housing, and coexistence regarding compliance.

## 1. Introduction

A healthy diet should provide the optimal amounts of energy and nutrients essential for life [1]. The European recommendations indicate that a balanced diet is based on an adequate energy contribution of each macronutrient, corresponding 10%–15% to proteins [2].

Previous studies conducted in the university population have shown a decrease in the consumption of fruits, vegetables, cereals, legumes, and fish, together with an increase in the consumption of meats, sweets, some snacks, and sugary drinks, which suggests a decrease in the adherence to the traditional Mediterranean diet (MD) in the young-adult population [3,4,5,6,7,8,9,10,11]. One of the main deviations of the MD pattern in the Spanish university population is the high consumption of meat [3,5,7]. Different meta-analyses show that a high consumption of red and processed meat can increase total mortality [12], different types of cancer [13,14,15,16], and cardiovascular diseases [17], which are currently considered one of the main causes of death in young adults [18]. This fact is aggravated, in addition, by the low adherence to the MD of the students [6].

The time period between late adolescence and early adulthood is an important time for the establishment of long-term behavior patterns [3,4,5,6,19]. Changes in the home and school/work environments, in social influences, in financial circumstances, and the establishment of partner relationships are also common [20,21,22]. There are even differences in diet inherent to the region or community where one lives, for example, between the north and the south of the same country [8]. Many of these factors have shown to be associated with diet and eating behaviors in this age range [20,21,22].

In this way, the university stage can be considered as “window of opportunity” for the promotion of healthy lifestyles among the adult population [3]. The objective of this study was to identify the sociodemographic characteristics associated with consumption of meat in first-year university students (UniHcos project).

## 2. Material and Methods

**Figure 1:**
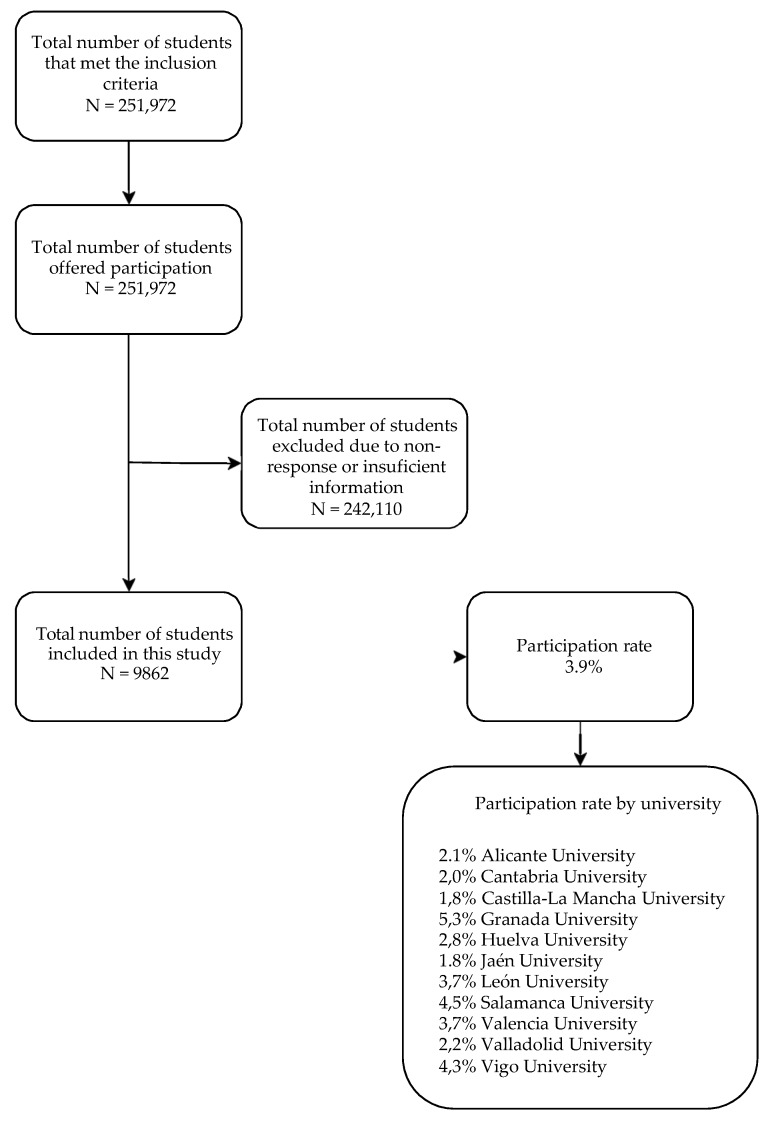
Recruitment flowchart (UniHcos project, 2018 Spain).

This cross-sectional study is framed within the UniHcos project, a multicenter study of multipurpose prospective cohorts involving 11 Spanish universities (León, Vigo, Jaén, Granada, Salamanca, Huelva, Alicante, Cantabria, Valladolid, Castilla-La Mancha, and Valencia) [4]; whose general objective is to know the lifestyle habits with which students enter university and their modification during their stay. The UniHcos project has the approval of the Ethics Committees of the collaborating universities and the integration of the information file in the Data Protection Agency complies with the Organic Law of Protection of Personal Data. The main inclusion criteria were to be a first-year student and first enrollment of all the grades of each of the participating universities. The information was collected through the completion of an online self-questionnaire completed by all students who met the selection criteria and agreed to participate in the project during the 2011–2018 academic years. Informed consent and ethical permission are also included in the online questionnaire. A flow chart detailing the recruitment process starting with the number of students who were offered participation and ending with the number of those that were finally included in this study is included (Figure 1). In the authors experience, low participation rates in research similar to that presented here is common among first-year university students. This carries with it a unit non-response bias, which appears when there exists a difference between the characteristics or responses of individuals invited to participate and those of the subjects finally included in the study. To avoid this as much as possible, the questionnaire was designed to try to avoid a design that would make it more likely for certain groups to participate or not in the study. It was sent via institutional email and students were given ample time and reminders to respond as well as assured confidentiality. Measuring and adjusting for non-response bias using weighting-class adjustments, poststratification, or propensity models was not possible due to the lack of sufficient demographic or database variables. Item non-response bias, which occurs when certain questions in a survey are not answered by a respondent, was not a concern as those students without sufficient information were excluded from the study.

The independent variables are related to the personal and sociodemographic characteristics and geographical location of the universities included in the UniHcos Project: Universities of the North (Cantabria, Castilla-La Mancha, León, Vigo, Salamanca, and Valladolid) and Universities of the South (Granada, Jaén, Huelva, Alicante, and Valencia).

The personal and sociodemographic variables were: Sex (male, female); age (years); body mass index (BMI) (˂18.5, 18.5–24.5, 25–30, >30) [23]; marital status (single, domestic partner, married, separated, divorced, widowed); employment status (only study and I do not look for work, study and I look for work, study and work part-time, study and work full-time); housing, defined as the place where students live during the course (home—family, residence—hall/residence—university, rental, home—own, others); and coexistence, defined as people with whom the student lives during the course (with my parents, roommates/friends, with my partner, with my children, alone).

For the interpretation of the data, the variables were re-categorized:

- Marital status: Single (single, separated, divorced, widowed), married (married, domestic partner);
- Employment status: Unemployed (only study and do not look for work, study and look for work), employed (study and work part time, study and work full time);
- Housing: Family home, university residence (residence hall/university residence), rental (rental, home-own, others);
- Coexistence: Parents, roommates (roommates/friends), partner (with my partner, with my children), alone.

The dependent variable was constructed from the answers to the food frequency consumption section (FFCS) of the online self-questionnaire completed by all students modeled after question 96 of Section H4 of the 2006 Spanish National Health Survey and which had been previously validated. The frequency of consumption of the following food groups was considered: (i) Consumption of fresh meat: Non-processed meat group (chicken, veal, pork, lamb, etc.), and (ii) the consumption of processed meats: Hamburgers, hot dogs, kebab and sausages, and cold meats [24]. The FFCS has 5 consumption frequency options from which to choose (daily, 3–4 times/week but non-daily, 1–2 times/week, <1 time/week, never/almost never). In this study, the 5 responses were regrouped into 4; specifically, the responses “daily” and “3–4 times/week but not daily” were group together into “≥3–4 times/week”. Within the fresh meat group, no distinction is made between white and red meat and therefore these data are not available in this study.

To assess the factors associated with consumption of fresh meat and processed meats, the recommendations established in the pyramid of the Mediterranean Diet Foundation 2010 (PFDM) were taken as gold standard, where the recommended consumption of white meat in a healthy adult population is defined as 2 servings/week, the consumption of red meat should be limited to <2 servings/week, and the consumption of processed meat must be <1 serving/week [25]. For the interpretation of the results, the frequencies of consumption were grouped according to “compliant” or “non-compliant” with the recommendations of the PFDM [25]. In addition, for fresh meat consumption, the “non-compliant” group was divided into underconsumption and overconsumption in order to determine if non-compliance is due to a deficient or excessive consumption of fresh meat and to be able to study those factors associated with each consumption level.

Since no separate data for white and red meat consumption were available, for the consumption of fresh meat, “compliant” was agreed among the authors to be defined as 1–2 times/week and the category “non-compliant” to include never/almost never +<1 time/week +≥3–4 times/week. While this is in fact an incorrect assumption for red meat since never/almost never and <1 times/week would also be in line with recommendations, after discussion it was deemed that defining “compliant” as 1–2 times/week would be the best solution to bridge the difference in the recommendations for white and red meat and the data available. For the consumption of processed meats, “compliant” corresponds to never/almost never +<1 time/week; the category “non-compliant” corresponds to 1–2 times/week +≥3–4 times/week.

The descriptive analysis is used for frequencies of consumption (absolute and relative) and was performed for the total sample, according to sociodemographic variables. Chi-square test with a statistical significance level of *p* < 0.05 was performed to check if there was a relationship between the groups.

The associated factors were analyzed using binary logistic regression (OR) techniques and 95% confidence intervals (CI) were calculated, stratified by university of origin. A multivariate analysis (ORa) adjusted for potential confounding factors was performed: Sex, BMI, marital status, employment status, housing and coexistence. The analysis of the data was performed with the statistical package IBM-SPSS version 20.0 (IBM Corp. Released 2011. IBM SPSS Statistics for Windows, Version 20.0. Armonk, NY, USA: IBM Corp.)

## 3. Results

Mean participation among all university students was 3.9% with a range between 1.8% and 5.3%. Within the characteristics of the study population, women represented 71.2% of the university population studied, with an average age of 20 years, (SD: 4.52), with universities of the north being 20.00 years (SD: 4.42) and those of the South being 20.18 (SD: 4.57), without significant difference (*p* > 0.05). A total of 361 (3.7%) students of those included in the study declared following a meat-free diet.

Table 1 describes the sociodemographic characteristics of the population studied according to geographical location. It was observed that the percentage or number of students who declare to live with their parents was greater than those who live in a rental apartment. Statistically significant differences were found (*p* < 0.001) between the percentage of students who reside in university residence, with those of the north (16.6%) being higher than the south of Spain (7.4%). Likewise, students who live alone in the universities of the north represented a higher percentage (11.7%) than the university students of the south (7.3%).

Of the population sample studied, 96.3% consumed some type of meat and 3.7% declared following a meat-free diet. Of the university students that consumed meat, 99.4% consumed fresh meat and 70.7% consumed processed meat. As a whole, 19.9% of the students complied with the recommendations for fresh meat consumption. If those following a meat-free diet are excluded, compliance rises to 20.7%. The recommendation for processed meat were met by 73.5% of the studied population and 76.3% met the requirements after the removal of non-meat eaters. Students that met recommendations for both fresh and processed meat consumption made up 3.8% of the sample as a whole and 3.9% when meat-free students are excluded.

Table 2 shows the fulfillment of the meat consumption recommendations by universities and according to their geographic distribution, in which it is noteworthy that only 16.0% of university students from the north and 22.7% from the south fulfilled the recommendations in the frequency of consumption of fresh meat; while 78.8% of university students from the north and 69.9% from the south complied with the frequency of consumption of processed meats, these results being statistically significant (*p* < 0.001).

Table 3 shows compliance with the recommendations for meat consumption according to sex, BMI, and sociodemographic variables. The results show differences between compliance with the recommendations in the consumption of both fresh meat and processed meat in relation to sex, employment status, and coexistence. Significant differences for compliance with the recommendations in the consumption of processed meat are found for BMI and significant differences for compliance with the recommendations in the consumption of fresh meat are found for housing. The majority of women and men did not comply with the recommendations for consumption of fresh meat but did comply with the recommendations for processed meat. However, more women met the recommendations for fresh meat while more men met the recommendations for processed meat. When separated according to BMI, the best compliance with recommendations for processed meat was found in the normal weight group followed by the obese, with overweight and underweight coming in last with very similar values. Regarding employment status, there was a higher rate of compliance with recommendations among those students that are employed. In relation to the housing, the rate of compliance with fresh meat recommendations was highest among the group that live in a rental or own home. In relation to the variable coexistence, the highest rate of compliance with recommendations was found in those that live with a partner. Meanwhile, the lowest rate of compliance with fresh meat recommendations was found in those that live alone and for processed meat recommendations it was found among those that live with roommates.

In Table 4, the level of and factors associated with compliance with recommendations on the frequency of fresh meat consumption according to sociodemographic characteristics of the university population are shown. When comparing students in the “compliant” and “insufficient” categories, males were more likely to meet recommendations than females (OR = 1.6; 95% CI: (1.2–2.0)), however, if the comparison is between the “compliant” and “excessive” categories, females wer more likely to meet the established recommendations (OR = 0.7; 95% CI: (0.6–0.8)). Those students that are married were less likely to comply with fresh meat consumption recommendations when the “compliant” and “insufficient” groups are compared (OR = 0.8; 95% CI: (0.6–1.0)). Differences depending on employment status appeared only for the “compliant”/“excessive” comparison with those employed more likely to meet recommendations (OR = 1.2; 95% CI: (1.0–1.4). For housing, differences appeared in both set of comparisons, “compliant”/“insufficient” and “compliant”/“excessive”. For the first comparison, students that live in a university residence were less likely to meet the recommendations of fresh meat consumption (OR = 0.5; 95% CI: (0.5–0.7)), however they were more likely to meet recommendations when the comparison is between “compliant” and “excessive” consumption (OR = 1.4; 95% CI: (1.2–1.5)). Students that live in rental/own home were also more likely to meet recommendations when the comparison is between “compliant” and “excessive” (OR = 2.3; 95% CI: (1.9–2.7)). In regards to coexistence, those students that live with roommates (OR = 0.4; 95% CI: (0.3–0.6)) or alone (OR = 0.6; 95% CI: (0.4–0.9)) were less likely to comply with fresh meat consumption recommendations when the “compliant” and “insufficient” groups are compared. However, when the “compliant” and “excessive” groups are compared, students that live with a partner (OR = 0.9; 95% CI: (0.7–1.0)) or alone (OR = 0.7; 95% CI: (0.6–1.0)) were less likely to comply with fresh meat consumption recommendations. No significant differences were observed with regards to BMI.

Table 5 shows the sociodemographic factors associated with compliance with the recommendations on the frequency of meat consumption, differentiating between northern and southern universities in Spain. In relation to sex, being a woman increased compliance with recommendations on the consumption of both fresh meat and processed meats without showing significant differences between northern (ORa = 1.4; 95% CI: (1.1–1.7)) (ORa = 1.3; 95% CI: (1.1–1.6)) and southern (ORa = 1.3; 95% CI: (1.1–1.5)) (ORa = 1.6; 95% CI: (1.4–1.8)) universities. Obesity (BMI > 30) appeared to increase compliance with the recommendations for fresh meat consumption in northern universities (ORa = 1.4; 95% CI: (1.1–1.7)). Employment only increased compliance with the recommendations for fresh meat consumption in southern universities (ORa = 1.2; 95% CI: (1.0–1.4)). Living in rental/own home increased compliance with the recommendations for fresh meat consumption in northern (ORa = 2.1; 95% CI: (1.6–2.8)) and southern (ORa = 1.8; 95% CI: (1.3–2.3)) universities without significant differences between them; while the probability of compliance with the recommendations on the consumption of processed meats was only favored in northern universities (ORa = 1.7; 95% CI: (1.3–2.1)). Regarding coexistence, living with a partner increasds compliance with processed meat recommendations in both sets of universities ((ORa = 1.3; 95% CI: (1.0–1.7)) (ORa = 1.2; 95% CI: (1.0–1.5)). Marital status did not appear to affect compliance with the recommendations on the consumption of fresh or processed meats in either group of universities.

## 4. Discussion

The results of the study show a lack of compliance with the recommendations of consumption of fresh meat in Spanish university students according to the standards of the recommendations of the MD. The finding that only 3.8% of the student population studied comply with the recommendations for both fresh and processed meat must be highlighted. The results also show that female employed students living in rental accommodations with a partner are more likely to meet the recommendations for consumption of fresh meats while male employed students living in rental accommodations with a partner are more likely to meet the recommendations for consumption of processed meats.

Given the change shown in the epidemiological profile of meat consumption and eating habits that are motivating a nutritional transition and distancing from the MD in university students, it is relevant to know the risk factors related to sociodemographic characteristics, which affect aspects related to eating lifestyles, physical activity, culinary aptitude, and shopping skills, in order to prioritize promotion strategies. Studies that provide information on associated factors, such as the practice of physical activity, culinary skills, habits, and shopping preferences, among others, related to the consumption of fresh and processed meat in the university population are needed.

The beginning of tertiary education is an important time in an individual’s life, as it often represents a period of increased responsibility regarding diet and lifestyle choices. However, usually at this time, young adults lack experience in food shopping, preparation, and meal planning. The choice and consumption of foods is influenced by psychological, social, cultural, economic, and biological factors [26]. The most commonly suggested circumstances that affect diet choices include changes in living arrangements [27], financial cost and resources [28], and the ever-increasing availability of convenience and/or fast foods [29].

The university stage is considered an opportunity for the promotion of healthy lifestyles among the adult population. Therefore, from a public health standpoint, it is necessary to prioritize health education strategies and other interventions that promote healthy habits in which the sociodemographic characteristics of the university population are considered. Public health guidelines should prioritize adequate meat consumption, especially of fresh meats in the university population, that encourage compliance with dietary patterns of MD. The latest survey in Spain on Consumption Habits 2017 [30] shows a change in eating habits especially in young consumers who prefer the purchase of fresh products compared to prepared and frozen dishes, possibly because governmental and private institutions have developed communication and advertising strategies to achieve more informed consumption.

The frequency of students adhering to the recommendations for consumption of meat throughout the country is 19.9% for fresh meat and 73.5% for processed meat. In relation to the adherence to the recommendations for consumption of meat, compliance is different throughout the territory depending on the type of meat studied, fresh vs. processed (Universities of the South: 22.7% and 69.9%, Universities of the North: 16.0% and 78.8%). Compliance is higher in the southern universities of Spain for fresh meat and in the northern universities for processed meat. While both regions share a very low compliance with the recommendations for fresh meat and an acceptable one for processed meat, the differences between the northern and southern universities suggest the existence of different diet patterns among the regions. It is interesting to know these data to promote specific interventions in the regions most in need, which would save time and money for the organizations.

There are several studies that analyze the differences in adherence to MD between the north and the south of the country, although they do not specify the differences between the different types of meat [7,9]. A study conducted in Italy shows that adherence to MD is higher in the south of the country [7]. Another study carried out by the University of Alicante shows that the regions of the south-east of the country (Comunidad-Valenciana (5.4%), Islas-Baleares (4.6%), and Andalucía (4.3%)) are the ones that present worse quality of diet [4].

Different studies carried out in the university population coincide with the results of the present work, although with smaller sample sizes, as they also reveal a meat consumption higher than the recommendations. A study carried out at the University of Alicante (2012) highlights that more than 90% of students have excessive consumption of red meat [10]. In terms of macronutrients, a study conducted at the University of Castilla-La Mancha shows that the consumption of protein from meat in the diet of students was 10% higher than the average of the general population (average consumption 198.2 g/day, 150 g/day women and 225 g/day men) [1]. At the European level, Ansari and collaborators (2012) show an excessive daily consumption of meat in half of the students of each country studied: Denmark 52.7%, Bulgaria 47.4%, Poland 46.3%, and Germany 44.2% [26], the results of this study being in Spanish university students above 80.1% in meat and approximately 26% in processed meat.

With regards to associated personal characteristics, statistically significant differences were found in the consumption of meat according to the sex of the students (*p* < 0.001). Different studies carried out both in Spain (University of Murcia [31]: Men 272 g/day, women 205 g/day, *p* < 0.000, University of Castilla-La Mancha [3]: Men 88.7 g/day, women 75, 3 g/day, *p* < 0.001), as in other countries (University of Malaysia [32]: Men 71.33 g/day, women 63.01 g/day, *p* = 0.004; universities in Germany, Denmark, Poland, and Bulgaria [26], *p* < 0.001) show the same differences, so that specific intervention strategies may be necessary according to sex in the students that help improve eating habits.

In addition, differences are observed in the probability of complying or not with the recommendations on the consumption of meat according to the sociodemographic characteristics of the university students. Thus, students living in a university residence are the least likely to meet the recommendations of meat consumption. These results are similar to those obtained in a Belgian university [33], which showed a negative association between the use of the canteen and healthy habits because the nutritional quality of the meals was low. On the contrary, different studies suggest that students who eat regularly in cafeterias have a lower protein consumption than students who eat elsewhere [34,35,36]. These contradictory results may reflect a lack of homogeneity in the quality of the menus offered by the different catering services, both in Spain and in other countries of the world. In this sense, six of the universities included in this study (León, Vigo, Salamanca, Huelva, Alicante, and Cantabria) belong to the Spanish Network of Healthy Universities (REUS). This network aims to strengthen universities as health promoting entities. Of the universities included in this study and in the REUS, only strategic lines and healthy eating programs have been found in the universities of Alicante, Castilla-La Mancha, and León [37,38,39]. Therefore, it could be advisable to conduct new studies to check the menus offered to students, offer dietary nutritional advice, and develop common healthy eating guidelines for its implementation in the different dining rooms, restaurants, bars, and vending machines of Spanish university campuses.

In relation to compliance with the recommendations for meat consumption and coexistence, significant differences are observed. No other studies have been found that relate this characteristic to general or specific meat consumption. Most of the studies found analyzed the effect of housing characteristics on the diet of university students differing between living at home with family or living outside the family home. There are studies that show a lower consumption of meat in students who do not live in the family home [11,25]. Thus, a European study shows that living outside the family home promotes lower consumption of meat, in all the countries studied (Germany, Denmark, Poland, and Bulgaria) (OR = 0.62; IC95% 0.52–0.75) [26].

Another study analyzing food acculturation in Greek students moving to Glasgow (Scotland) shows that consumption of red meat decreased in students after moving in, (frequency of weekly consumption (FCS) before = 3.6; FCS after = 2.2; *p* = 0.002), although this difference is not found when students stay in their country of origin (FCS before 3.9, FCS after 3.6, *p* = 0.695) [11]. The authors explain these results due to the proximity of the study to the outbreak of Bovine Spongiform Encephalopathy that emerged in the United Kingdom in 1996, which led to a rejection of meat consumption in later years (the data were collected in 2000). On the other hand, several studies conducted in the United States and Greece claim not to find significant differences between the consumption of meat and living in the family home or outside it (*p* > 0.5), although they do not measure who the students live with partners [40,41]. Only significant differences have been found in the consumption of pork meat according to the students lived on campus at university (*n* = 72), off-campus (*n* = 68), or at home with family (*n* = 4) (*p* < 0.001) but not in the other types of meat [31]. No studies have been found that show an increase in meat consumption when students do not live in the family home, an interesting fact considering that there are studies that show a general worsening of eating habits (lower consumption of fruits and vegetables, lower consumption of homemade food, among others) in students who live outside the family home [11,26,40].

### Limitations

One of the limitations of this study is the design (transversal), therefore the results must be interpreted with caution. The questionnaire used to collect the information, a self-completed FFCS, could present a possible bias of social desirability in terms of wanting to indicate the consumption of foods that young people consider to have better or healthier characteristics. However, it is possible that this bias was controlled because the user had to concentrate on determining the number of times he/she consumes a type of meat and not on its healthy characteristics. In addition, FFCS is one of the most used questionnaires in population studies, to obtain information on the times or frequency with which a specific food is consumed in order to compare it with nutritional recommendations. Another limitation is the participation rate of the study, which is below 4%. Participation in the study was completely voluntary and invitations were sent by way of email to the students’ academic email accounts. These two factors could have influenced the low participation as first year university students may not prioritize taking part in such studies and/or may not utilize the academic emails often.

## 5. Conclusions

The results of the study show a lack of compliance with the recommendations for consumption of fresh meat in Spanish university students according to the standards of the recommendations of the MD and an acceptable level of compliance with the recommendations for consumption of processed meat. Compliance is different throughout the territory depending on the type of meat studied, fresh vs. processed, with it being higher in the southern universities of Spain for fresh meat and in the northern universities for processed meat. The results also show an association with sex, employment, housing, and coexistence regarding compliance. Recommendations for consumption of fresh meat are more likely to be met by female employed students living in rental accommodations with a partner, meanwhile the recommendations for consumption of processed meat are more likely to be met by male employed students living in rental accommodations with a partner.

## Figures and Tables

**Table 1:** Sociodemographic characteristics of the university sample according to the geographical location of the universities.

	Total	Universities of the North ^a^	Universities of the South ^b^	*p* ^c^
	*n* (%)	*n* (%)	*n* (%)	
Total	9862 (100)	3982 (100)	5880 (100)	
Marital status				
Single	9002 (91.4)	3662 (92.1)	5340 (90.9)	0.019
Married	852 (8.6)	315 (7.9)	537 (9.1)	
Employment status				
Unemployed	8821 (89.4)	3575(89.8)	5246 (89.2)	0.196
Employed	1041 (10.6)	407 (10.2)	634 (10.8)	
Housing				
Family home	4484 (45.5)	1705 (42.8)	2779 (47.3)	<0.001
University residence	1101 (11.2)	663 (16.6)	438 (7.4)	
Own home	270 (2.7)	109 (2.7)	161 (2.7)	
Other	136 (1.4)	76 (1.9)	60 (1.0)	
Rental	3871 (39.3)	1429 (35.9)	2442 (41.5)	
Coexistence				
Parents	4516 (45.8)	1760 (44.2)	2756 (46.9)	<0.001
Roommates	4042 (41.0)	1604 (40.3)	2438 (41.5)	
Partner	432 (4.4)	173 (4.3)	259 (4.4)	
Alone	870 (8.8)	443 (11.7)	427 (7.3)	

^a^ Universities of the North: Cantabria, León, Vigo, Salamanca, and Valladolid. ^b^ Universities of the South: Granada, Jaén, Huelva, Alicante. ^c^ *p*-Value obtained through the Chi-square test.

**Table 2:** Compliance with the recommendations on the frequency of consumption of fresh meats and processed meats by universities and according to geographical distribution.

		Compliance with Consumption Recommendations			
		Meat-Fresh ^a^		Meat-Processed ^b^	
	Total	Yes		Yes	
	n (%)	n (%)	*p* ^c^	n (%)	*p* ^c^
Total	9862 (100)	1969 (19.9)		7247 (73.5)	
Universities					
Alicante	788 (8.0) (100)	153 (7.8) (19.4)	<0.001	573 (7.9) (72.7)	<0.001
Cantabria	85 (0.9) (100)	13 (0.7) (15.3)		64 (0.9) (75.3)	
Castilla-La Mancha	159 (1.6) (100)	25 (1.3) (15.7)		119 (1.6) (74.8)	
Granada	2930 (29.7) (100)	710 (36.1) (24.2)		2038 (28.1) (69.6)	
Huelva	427 (4.3) (100)	137 (7.0) (32.1)		285 (3.9) (66.7)	
Jaén	288 (2.9) (100)	80 (4.1) (27.8)		195 (2.7) (67.7)	
León	828 (8.4) (100)	133 (6.8) (16.1)		662 (9.1) (80.0)	
Salamanca	1097 (11.1) (100)	121 (6.1) (11.0)		834 (11.5) (76.0)	
Valencia	1447 (14.7) (100)	253 (12.8) (17.5)		1019 (14.1) (70.4)	
Valladolid	616 (6.2) (100)	72 (3.7) (11.7)		468 (6.5) (76.0)	
Vigo	1197 (12.1) (100)	272 (13.8) (22.7)		990 (13.7) (82.7)	
Universities North/South distribution					
North	3982 (40.4) (100)	636 (32.3) (16.0)	<0.001	3137 (43.3) (78.8)	<0.001
South	5880 (59.6) (100)	1333 (67.7) (22.7)		4110 (56.7) (69.9)	

^a^ Recommendations for the consumption of fresh meat according to the Dietary Guidelines of the Mediterranean Diet Foundation 2010: 1–2 times/week. ^b^ Recommendations for the consumption of processed meats according to the Food Guide of the Mediterranean Diet Foundation 2010: <1 time/week. ^c^ *p*-value obtained through the Chi-square test.

**Table 3:** Compliance with recommendations on the frequency of meat consumption according to personal and sociodemographic characteristics of the university population.

		Compliance with Consumption Recommendations			
		Meat-Fresh ^a^		Meat-Processed ^b^	
	Total	Yes		Yes	
	*n* (%)	*n* (%)	*p* ^c^	*n* (%)	*p* ^c^
Total	9862 (100)	1969 (19.9)		7247 (73.5)	
Sex					
Male	2737 (27.8) (100)	452 (23.0) (16.5)	<0.001	1859 (25.7) (75.6)	<0.001
Female	7125 (72.2) (100)	1517 (77.0) (21.3)		5388 (74.3) (67.9)	
BMI					
Underweight (˂18.5)	996 (10.1) (100)	197 (10.0) (19.8)	0.365	710 (9.8) (71.2)	0.039
Normal weight (18.5–24.5)	7100 (72.0) (100)	1396 (70.9) (19.7)		5262 (72.6) (74.1)	
Overweight (25–30)	1429 (14.5) (100)	301 (15.3) (21.1)		1022 (14.1) (71.5)	
Obesity (>30)	345 (3.5) (100)	75 (3.8)21.7)		253 (3.5) (73.3)	
Marital status					
Single	9002 (91.4) (100)	1795 (91.2) (19.9)	0.414	6613 (91.3) (73.5)	0.427
Married	852 (8.6) (100)	173 (8.8)20.3)		629 (8.7) (73.8)	
Employment status					
Unemployed	8821 (89.4) (100)	1731 (87.9) (19.6)	0.008	6458 (89.1) (73.2)	0.039
Employed	1041 (10.6) (100)	238 (12.1) (22.9)		789 (10.9) (75.8)	
Housing					
Family home	4484 (45.5) (100)	856 (43.5) (19.1)	<0.001	3274 (45.2) (73.0)	0.187
University residence	1101 (11.2) (100)	137 (7.0) (12.4)		793 (10.9) (72.0)	
Rental/Own home	4277 (43.4) (100)	976 (49.6) (22.8)		3180 (43.9) (74.4)	
Coexistence					
Parents	4516 (45.8) (100)	849 (43.1) (18.8)	0.003	3296 (45.5) (73.0)	0.004
Roommates	4042 (41.0) (100)	852 (43.3) (21.1)		2945 (40.6) (72.9)	
Partner	432 (4.4) (100)	106 (5.4) (24.5)		345 (4.8) (79.9)	
Alone	870 (8.8) (100)	162 (8.2) (18.6)		661 (9.1) (76.0)	

^a^ Recommendations for fresh meat consumption according to the Pyramid Mediterranean Diet Foundation 2010: 1–2 times/week, ^b^ Recommendations for the consumption of processed meats according to the Pyramid Mediterranean Diet Foundation 2010: <1 time/week, ^c^ *p*-Value obtained through the Chi-square test (per columns).

**Table 4:** Level of and factors associated with compliance with recommendations on the frequency of fresh meat consumption according to personal and sociodemographic characteristics of the university population.

		Compliance with Fresh Meat Consumption Recommendations ^a^				
		Compliant Consumption (CC)	Insufficient Consumption (IC)		Excessive Consumption (EC)	
	Total	(1–2 Times/Week)	(Never/Almost Never + <1 Time/Week)	(CC/IC) OR (95%CI)	(≥3–4 Times/Week)	(CC/EC) OR (95%CI)
	n (%)	*n* (%)	*n* (%)		*n* (%)	
Total	9862 (100)	1969 (19.9)	703 (7.1)		7190 (72.9)	
Sex						
Male	2737 (100)	452 (16.5)	113 (4.1)	1.6 (1.2–2.0)	2172 (79.4)	0.7 (0.6–0.8)
Female	7125 (100)	1517 (21.3)	590 (8.3)	Ref.	5018 (70.4 )	Ref.
BMI						
Underweight (˂18.5)	996 (100)	197 (19.8)	84 (8.4)	0.9 (0.6–1.4)	715 (71.8)	1.2 (0.9–1.5)
Normal weight (18.5–24.5)	7100 (100)	1396 (19.7)	498 (7.0)	Ref.	5206 (73.3)	Ref.
Overweight (25–30)	1429 (100)	301 (21.1)	91 (6.4)	1.0 (0.7–1.8)	1037 (72.5)	1.1 (0.8–1.5)
Obesity (> 30)	345 (100)	75 (21.7)	30 (8.5)	0.8 (0.5–1.3)	240 (69.7)	1.1 (0.8–1.4)
Marital status						
Single	9002 (100)	1795 (19.9)	624 (6.9)	Ref.	6583 (73.1)	Ref.
Married	852 (100)	173 (20.3)	78 (9.2)	0.8 (0.6–1.0)	601 (70.5)	1.1 (0.9–1.3)
Employment status						
Unemployed	8821 (100)	1731 (19.6)	628 (7.1)	Ref.	6462 (73.3)	Ref.
Employed	1041 (100)	238 (22.9)	75 (7.2)	1.2 (0.9–1.5)	728 (69.9)	1.2 (1.0–1.4)
Housing						
Family home	4484 (100)	856 (19.1)	208 (4.6)	Ref.	3420 (76.3)	Ref.
University residence	1101 (100)	137 (12.4)	58 (5.3)	0.5 (0.5–0.7)	906 (82.3)	1.4 (1.2–1.5)
Rental/Own home	4277 (100)	976 (22.8)	437 (10.2)	0.9 (0.7–1.3)	2864 (67.0)	2.3 (1.9–2.7)
Coexistence						
Parents	4516 (100)	849 (18.8)	201 (4.5)	Ref.	3466 (76.7)	Ref.
Roommates	4042 (100)	852 (21.1)	383 (9.5)	0.4 (0.3–0.6)	2807 (69.4)	1.1 (0.9–1.3)
Partner	432 (100)	106 (24.5)	32 (7.4)	0.8 (0.6–1.1)	294 (68.1)	0.9 (0.7–1.0)
Alone	870 (100)	162 (18.6)	87 (10.0)	0.6 (0.4–0.9)	621 (71.4 )	0.7 (0.6–1.0)

^a^ Recommendations for fresh meat consumption according to the Pyramid Mediterranean Diet Foundation 2010: 1–2 times/week.

**Table 5:** Factors associated with compliance with nutritional recommendations in the frequency of consumption of fresh meat and processed meats, according to geographical region.

	Compliance with Fresh Meat Consumption Recommendations					Compliance with Processed Meat Consumption Recommendations				
	Universities of the North ^a^		Universities of the South ^b^			Universities of the North ^a^		Universities of the South ^b^		
	OR_Crude_^c^	OR_Adjusted_^d^	OR_Crude_^c^	OR_Adjusted_^d^	*p* ^e^	OR_Crude_^c^	OR_Adjusted_^d^	OR_Crude_^c^	OR_Adjusted_^d^	*p* ^e^
	(95% CI)	(95% CI)	(95% CI)	(95% CI)		(95% CI)	(95% CI)	(95% CI)	(95% CI)	
Sex										
Male	1 Ref.	1 Ref.	1 Ref.	1 Ref.		1 Ref.	1 Ref.	1 Ref.	1 Ref.	
Female	1.4 (1.1–1.7)	1.4 (1.1–1.7)	1.3 (1.1–1.5)	1.3 (1.1–1.5)	0.677	1.3 (1.1–1.6)	1.3 (1.1–1.5)	1.6 (1.4–1.8)	1.6 (1.4–1.8)	0.073
BMI										
Under weight (<18.5)	1.2 (0.7–1.8)	1.2 (0.8–2.0)	1.3 (0.8–1.5)	1.1 (0.8–1.6)	0.647	1.0 (0.6–1.4)	1.0 (0.6–1.5)	1.1 (0.8–1.5)	1.0 (0.8–1.5)	- ^f^
Normal weight (18.5–24.5)	1 Ref.	1 Ref.	1 Ref.	1 Ref.		1 Ref.	1 Ref.	1 Ref.	1 Ref.	
Overweight (25–30)	1.0 (0.6–1.7)	1.1 (0.6–1.9)	1.2 (0.8–1.7)	1.2 (0.8–1.7)	0.642	1.1 (0.7–1.7)	1.2 (0.7–1.9)	1.2 (0.8–1.7)	1.2 (0.8–1.7)	0.993
Obesity (>30)	0.9 (0.6–1.5)	1.4 (1.1–1.7)	1.1 (0.8–1.5)	1.1 (0.8–1.6)	0.183	1.1 (0.7–1.8)	1.2 (0.8–1.9)	1.2 (0.8–1.7)	1.2 (0.8–1.7)	0.993
Marital status										
Single	1 Ref.	1 Ref.	1 Ref.	1 Ref.		1 Ref.	1 Ref.	1 Ref.	1 Ref.	
Married	1.3 (1.0–1.8)	0.8 (0.6–1.0)	1.4 (1–2)	1.2 (0.9–1.5)	0.781	1.3 (1.0–1.8)	1.1 (0.8–1.5)	0.9 (0.7–1.1)	0.9 (0.8–1.2)	0.950
Employment status										
Unemployed	1 Ref.	1 Ref.	1 Ref.	1 Ref.		1 Ref.	1 Ref.	1 Ref.	1 Ref.	
Employed	0.8 (0.6–1.1)	0.9 (0.7–1.1)	1.1 (0.8–1.4)	1.2 (1.0–1.4)	0.290	1.1 (0.8–1.4)	0.9 (0.7–1.1)	0.8 (0.6–0.9)	1.2 (1.0–1.5)	0.154
Housing										
Family home	1 Ref.	1 Ref.	1 Ref.	1 Ref.		1 Ref.	1 Ref.	1 Ref.	1 Ref.	
University residence	1.4 (1.2–1.7)	1.3 (0.9–1.9)	1.2 (1.1–1.3)	1.0 (0.7–1.3)	- ^f^	1.1 (0.9–1.3)	1.1 (0.8–1.6)	1.1 (1.0–1.2)	1.0 (0.8–1.4)	- ^f^
Rental/Own home	2.1 (1.6–2.8)	2.1 (1.6–2.8)	1.7 (1.3–2.3)	1.8 (1.3–2.3)	0.277	1.5 (1.2–1.9)	1.7 (1.3–2.1)	1.0 (0.8–1.2)	1.0 (0.8–1.3)	- ^f^
Coexistence										
Parents	1 Ref.	1 Ref.	1 Ref.	1 Ref.		1 Ref.	1 Ref.	1 Ref.	1 Ref.	
Roommates	1.1 (0.8–1.4)	1.2 (0.8–1.8)	1.1 (0.8–1.3)	1.3 (0.9–1.8)	0.703	1.0 (0.8–1.3)	1.2 (0.8–1.8)	1.2 (0.9–1.5)	1.2 (0.8–1.6)	0.993
Partner	0.9 (0.7–1.2)	1.2 (0.8–1.6)	0.9 (0.7–1.1)	1.0 (0.8–1.4)	- ^f^	1.1 (0.8–1.4)	1.3 (1.0–1.7)	1.2 (0.9–1.5)	1.2 (1.0–1.5)	0.138
Alone	0.6 (0.4–0.8)	0.9 (0.6–1.5)	0.9 (0.6–1.2)	1.0 (0.7–1.5)	- ^f^	1.0 (0.6–1.5)	1.3 (0.8–2.0)	0.7 (0.5–0.9)	0.7 (0.5–1.0)	0.002

^a^ Universities of the North: Cantabria, León, Vigo, Salamanca, and Valladolid. ^b^ Universities of the South: Granada, Jaén, Huelva, Alicante. ^c^ OR and IC (95%) obtained through binary logistic regression. ^d^ OR adjusted for all the independent variables included in the model (sex, BMI, marital status, employment status, housing, coexistence). ^e^ *p*-Value for OR adjusted models obtained through the Student’s *t*-test. ^f^ *p*-Value not available when OR = 1.0, however, confidence interval analysis suggests no statistical difference.

## Abbreviations

Adjusted odds ratio (ORa); Food frequency consumption section (FFCS); Frequency of weekly consumption (FCS); Mediterranean diet (MD); Odds ratio (OR); Pyramid of the Mediterranean Diet Foundation 2010 (PFDM); Spanish Network of Healthy Universities (REUS); Standard deviation (SD).
